# The Right Tool for the Job: A Review of Insect Mouthparts as a Tool Kit for Biomimetic Studies

**DOI:** 10.3390/biomimetics10040196

**Published:** 2025-03-24

**Authors:** Matthew S. Lehnert, Kendall O. Myers, Kristen E. Reiter

**Affiliations:** 1 Department of Biological Sciences, Kent State University at Stark, North Canton, OH 44720, USA; kmyers58@kent.edu; 2 Biology Department, Cuyahoga Community College, Highland Hills, OH 44122, USA; kristen.reiter@tri-c.edu

**Keywords:** mandibulate mouthparts, haustellate mouthparts, insect morphology, feeding mechanisms, structure–function relationships

## Abstract

Few traits exhibit a more diverse collection of exemplary structure–function relationships than the mouthparts of insects. The global dominance of insects is attributed to their diverse food sources, which are matched by an array of morphological and chemical adaptations: a ‘tool kit’ for biomimicry. This review provides an overview of insect mouthparts that have contributed to biomimetics, including information about morphology and functionality in relation to particular feeding mechanisms. Themes in the groups of insects employed for particular biomimetic studies, including their lineages and feeding strategies, are identified along with suggestions for future studies, which together underscore the importance and promise of the development of novel engineered devices inspired by the unique ‘tools’ of insect mouthparts.

## 1. Introduction

**Figure 1:**
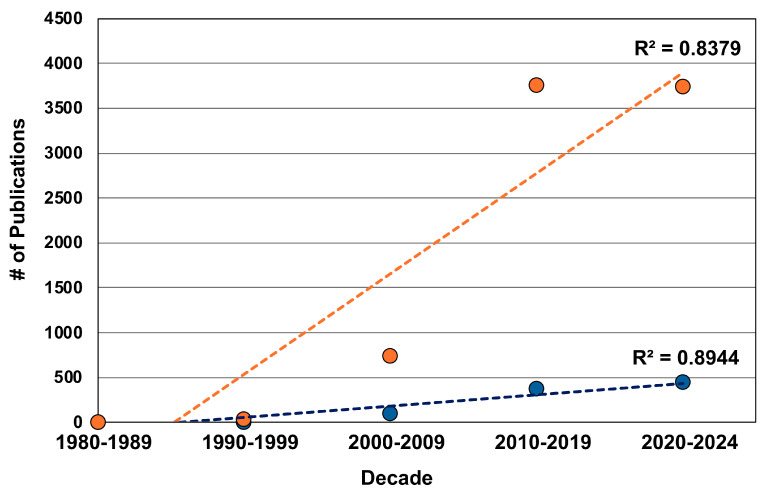
General growth of biomimicry of insects and insect mouthparts as determined by publication quantity per decade. The orange markers represent the number of publications using the keywords “Insect biomimicry” in Google Scholar. The blue markers represent a search using the keywords “Insect mouthparts biomimicry”. Both topics are experiencing growth, as indicated by the increasing number of publications.

The enormous diversity of insects and their corresponding adaptations provide a rich source of opportunities for the development of human-engineered analogs. The field of biomimetics (including bioinspiration for this review) is densely populated by studies motivated by insect adaptations; indeed, insects are arguably the ‘model group’ for biomimicry. Biomimetic studies that feature insects span multiple scales and lineages, providing inspiration for the development of flying robots [1,2,3], energy-efficient architecture [4,5,6], iridescent materials [7,8,9], anti-adhesive surfaces [10,11,12], optic probes [13,14,15], and cuticle-like materials [16,17,18], among others. Although several components of insect morphology, physiology, chemistry, and behavior have been applied to biomimetics, some structural complexes exhibit a spectrum of diverse adaptations and, in turn, create a “tool kit” to select from for an array of biomimetic studies. Insect mouthparts are an excellent example of a structural complex that serves as a tool kit for biomimicry. Researchers have exploited this tool kit for several applications, including improvements in cutting efficiency by studying the rate of curvature of mouthparts to reduce cutting forces, innovations in robotics by mimicking the mouthpart shape and axial-rotation abilities for gripping and transporting delicate objects, and the development of small needles that facilitate tissue penetration without buckling, to name a few—and the field is growing (Figure 1).

**Figure 2:**
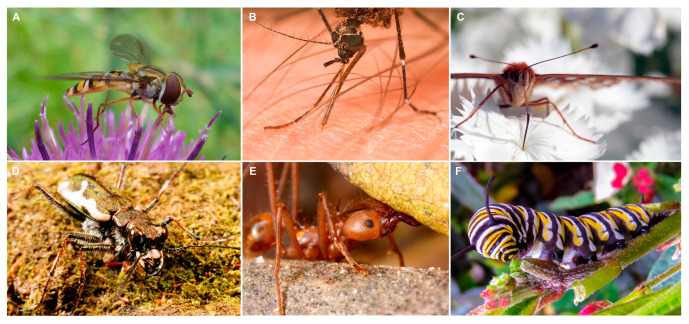
Diversity of insect feeding habits. (**A**) Image of a hoverfly, *Allograpta obliqua* (Diptera: Syrphidae) feeding from a flower with mouthparts adapted for fluid uptake. (**B**) The mosquito, *Ochlerotatus notoscriptus* (Diptera: Culicidae), piercing human skin for a blood meal. (**C**) A great spangled fritillary, *Speyeria cybele* (Lepidoptera: Nymphalidae) using its proboscis to feed from fluids inside a flower. (**D**) Image of a tiger beetle, *Neocicindela tuberculata* (Coleoptera: Cicindelidae) showing its mandibles adapted for biting and handling prey. (**E**) A leaf cutter ant, *Atta* sp. (Hymenoptera: Formicidae) using its mandibles to transport a leaf. (**F**) A larva of a monarch butterfly, *Danaus plexippus* (Lepidoptera: Nymphalidae) using its mandibles to feed on milkweed. (**A**) Image was acquired by John Chapman and used under the CC Attribution-Share Alike 4.0 International license. (**B**) Image is used under the CC Attribution-Share Alike 3.0 Unported license and was acquired by J.J. Harrison. (**C**) Image acquired by Sharon Mollerus and used under the CC Attribution 2.0 Generic. (**D**) Image used under CC Attribution-Share Alike 4.0 International license and acquired by John Marris. (**E**) Image was acquired by Malin Björnsdotter Åber and used under the CC Attribution-Share Alike 3.0 Unported license. (**F**) Image was used under the CC Attribution-Share Alike 3.0 Unported license and acquired by Ser Marr.

Insects are ubiquitous, and their ecological and evolutionary successes are largely linked to their ability to acquire nutrients from diverse sources in the environment, which involves a range of insect feeding strategies paralleled by a spectrum of mouthpart shapes and functionalities. Insect mouthparts represent an important interface between the insect and the environment and are adapted to handle and transport specific food items that span a range of material properties, including solid materials of varying hardness (e.g., leaves, wood, small arthropods, and stored products such as cereal or dog food) [19,20,21] and both Newtonian and non-Newtonian fluids [22,23,24] (Figure 2). In addition, the functionality of insect mouthparts is essential to many ecological services, such as pollination [25,26,27], seed dispersal [28], decomposition [29], and maintenance of predator-prey balances [30,31,32].

The wide range of mouthpart functionalities provides opportunities for biomimetic studies, which are further facilitated by the fact that insect mouthparts represent one of the most well-studied animal structures [33,34]. Extensive knowledge about insect mouthpart morphology provides some convenience for use in biomimicry because much of this field relies on structural adaptations. It is the coupling of the different morphologies and material properties that have allowed insects to successfully feed on an array of food sources while overcoming many physical challenges, thus providing motivation for the development of a range of novel devices that also perform different functions.

## 2. Ancestral Mouthpart Condition and Structural Mouthpart Interactions

The class Insecta, along with proturans, diplurans, and collembolans, comprises the monophyletic subphylum Hexapoda [35,36]. Several traits distinguish the insects from the non-insect hexapods, but one of the most fundamental is linked to mouthpart morphology—non-insect hexapods have entognathous mouthparts and monocondylic mandibles, whereas most insects are ectognathous and have dicondylic mandibles [37,38,39]. Entognathous mouthparts remain inside the head and the monocondylic mandibles are capable of rotating on multiple axes during feeding. The added rotational freedom might be adaptive for feeding on soft organic materials, such as leaf litter or fungi, but it prevents the ability to generate high bite forces needed for mastication.

**Figure 3:**
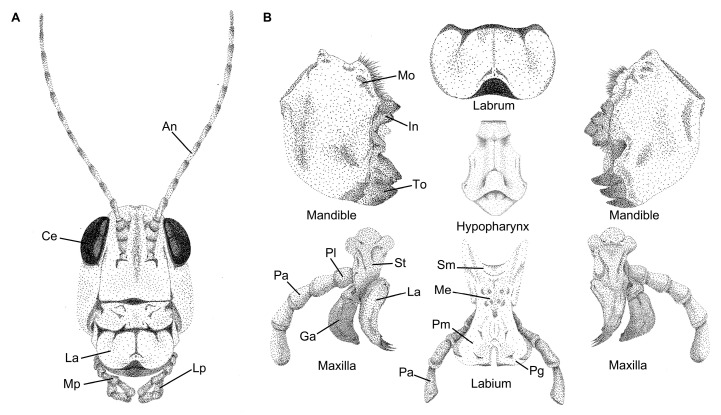
Illustrations of the head and mouthparts of the lubber grasshopper, *Romalea microptera* (Orthoptera), showing the plesiomorphic mouthpart condition. (**A**) Anterior view of the head showing the labrum (La), maxillary palpi (Mp), and labial palpi (Lp) mouthparts along with the compound eyes (Ce) and antennae (An) for reference. (**B**) Detailed illustrations of the different mouthparts, including the single labrum, pair of mandibles, hypopharynx, pair of maxillae, and the labium. The mandible shows three different regions, including the molar (Mo), incisor (In), and tooth (To) regions. The maxilla is composed of several parts, including the stipes (St), lacinia (La), galea (Ga), and of the maxillary palpi, the palpifer (Pl), and palpus (Pa). The labium also consists of several parts, including the submentum (Sm), mentum (Me), prementum (Pm), paraglossa (Pg), and a palpus (Pa). All illustrations were drawn by Erin Kelly.

Entognathous and ectognathous hexapods last shared a common ancestor approximately 400 mya; early insect lineages already had a mouthpart composition that consisted of the same structures observed among extant taxa [38]. It is well accepted that most insect mouthparts are derived from ancestral leg-like appendages, as determined from morphological and molecular studies [40,41,42], which produced the ancestral insect mouthpart assemblage known as mandibulate mouthparts. Evidence indicates that this plesiomorphic condition consisted of several structures, including a single labrum, two mandibles, two maxillae, a single labium, and a hypopharynx [33,43,44] (Figure 3). Each structure serves a different role in the ingestion process, which also relates to the preferred food source, providing some of the most noteworthy examples of convergent evolution [45,46,47] and structure–function relationships, cornerstones of evolutionary biology [48,49,50].

Labrum: Serves as an upper lip that facilitates the handling of food and closes the preoral cavity [40]. The evolutionary origins of the labrum are still being resolved, but unlike other insect mouthparts, evidence indicates that the labrum did not evolve from a leg-like appendage [51].
Mandibles: Facilitates the biting, handling, and mastication of food. Mandibles might consist of a tooth region, an incisor region, and a molar region, depending on the feeding habit of the insect [19,52,53]. In addition, because the mandibles are dicondylic with two points of articulation, they tend to move along a medial-lateral axis.
Maxillae: Provides several different functions, including assistance in handling food and gathering chemosensory information, and might aid in the mastication of food along with the mandibles [41]. The maxillae consist of several subcomponents, including a cardo and stipes that aid in articulation, and the lacinia and galea endites that extend from the maxillary palpus.
Labium: Serves as the lower lip and provides a means to close the preoral cavity. Similar to the labrum, the labium assists in food handling and the determination of food suitability. The labium is a fused pair of appendages and consists of several subcomponents, including the gula, postmentum, and prementum. The labium also might have labial palpi that are similar in functionality to the maxillary palpi [34].
Hypopharynx: Assists in moving food during the mastication process and functions similarly to a tongue, possessing a host of chemosensillae to determine food suitability [54].

These structures and their relative positioning to each other represent the ground plan for insect mouthparts (Figure 3); however, further modifications have provided paths to new modes of feeding and new sources for nutrient acquisition. Over evolutionary time, some structures fused together to take on new functions via the process of structural mouthpart interactions (SMIs), an apomorphy of insects [37]. Due to SMIs, novel morphologies, such as haustellate (fluid-feeding) mouthparts, evolved and provided access to new sources of nutritive liquids through the combination of conduits and a sucking pump in the head [47,55,56].

The purpose of this review is to summarize the contributions of insect mouthparts to biomimetics. We have organized our review according to different insect feeding categories as previously determined by mouthpart morphology and functionality [57], featuring the biting-chewing feeding mechanism under the mandibulate mouthparts category, and the piercing-sucking and nonpiercing-sucking (including sponge-sucking and lapping-sucking) feeding mechanisms under the haustellate mouthparts category. Although other insect feeding strategies exist, such as filter-feeding by black fly larvae (Simuliidae) [58] and the biting-sucking feeding mechanism of antlion larvae [59], we are not aware of their use in biomimetic studies; however, they might be useful for future studies. In addition, we focus most of this review on studies that included the production of a biomimetic tool, not only mentioning the possible applications.

## 3. Biomimicry of Insects with Mandibulate Mouthparts

### 3.1. Overview

The mandibulate mouthparts are the most widely represented mouthpart type among the 29 extant insect orders, where 26 orders (approximately 90%) have this configuration during at least one stage of their development; only the Thysanoptera, Hemiptera, and Phthiraptera (sucking lice) completely lack this mouthpart configuration [34,60,61]. In this section, we provide examples of insect-inspired biomimetic studies that feature lineages that retain the mandibulate-type mouthparts and a biting-chewing feeding mechanism throughout their development (immature and adult life stages), including hemimetabolous lineages (incomplete metamorphosis), such as grasshoppers, crickets, and katydids (Orthoptera), and dragonflies and damselflies (Odonata), and holometabolous lineages (complete metamorphosis), including beetles (Coleoptera) and ants, bees, and wasps (Hymenoptera). Despite additionally having a lapping-sucking feeding mechanism for nectar feeding, we included the western honey bee, *Apis mellifera* (Apidae), in this section because of the emphasis on mandible functionality. Some lineages with mandibulate mouthparts, such as antlions and lacewings (Neuroptera) and predaceous diving beetles (Coleoptera), have evolved conduits in their mandibulate mouthparts that operate in coordination with a sucking pump [34,59] and exhibit a biting-sucking feeding mechanism [62], but we are unaware of any biomimetic studies motivated by this mouthpart configuration.

### 3.2. Biomimicry with Biting-Chewing Mouthparts

Insects with a biting-chewing feeding mechanism have mandibles that cut and masticate food before ingestion. Species display adaptations that relate to particular food sources, such as those that use their mandibles to chew or cut through hard materials might have cuticle enriched with transition metals (zinc, iron, copper, and manganese), which creates sacrificial bonds and increases cuticle hardness [57,63,64]. In addition, mandibles might have regions of different morphologies, which serve different adaptive roles, including distal tooth, intermediate incisor, and proximal molar regions [65,66,67]. Predatory insects tend to have mandibles that are sharp at the tip with an elongated tooth region for capturing, piercing, and mashing prey [68,69,70], and herbivorous insects have mandibles that are augmented with well-defined molar regions for grinding tough plant material [71,72,73]. The diverse morphology of mandibles provides a tool kit from which we can choose to develop an array of biomimetic tools with cutting, gripping, anti-adhesion, and other functions.

#### 3.2.1. Mandible-Inspired Cutting Tools

**Figure 4:**
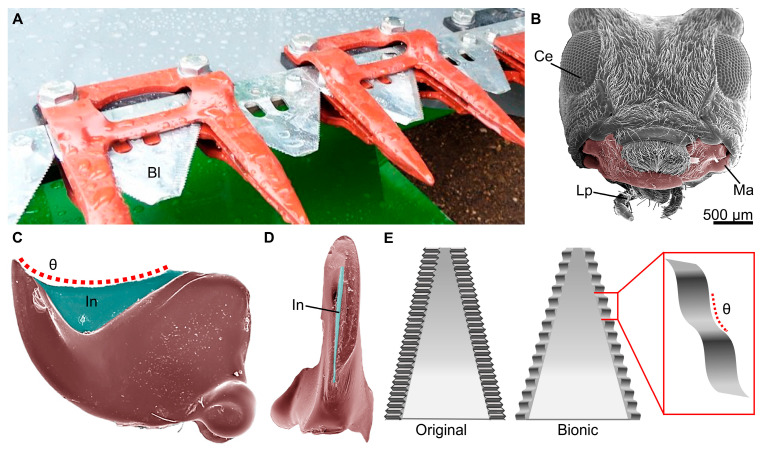
Representative biomimetic hemp harvest blades inspired by the mandibles of longhorn beetles. (**A**) An image of bionic hemp cutting blades (Bl) on an agricultural harvester that was improved through biomimicry. (**B**) A scanning electron microscopy (SEM) image of the head of the flat faced longhorn beetle, Acanthocinus obsoletus, a closely related species to Leiopus nebulosus, which had its mandibles used as inspiration for the new blade. The image shows the compound eye (Ce), labial palpi (Lp), and mandibles (Ma, false colored) similar to those used for biomimetic studies. (**C**) A false-colored SEM image of the ventral side of the mandibles of A. obsoletus. The incisor (In) region is false-colored blue, and the red dotted line represents the contour angle (*θ*) used to develop the serration pattern of the bionic blades. (**D**) False-colored SEM image showing the sharp cutting edge of the incisor on the medial side of the mandibles of A. obsoletus. (**E**) Schematic showing a representative original (left) and bionic (right) hemp harvesting blade with the different serration patterns. The bionic blades use the same contour angle (*θ*, shown in the red box) as the mandibles of *L. nebulosus* (based on [78]). The image in (**A**) was used with permission by Forever Green Worldwide Corporation at Hempcutter.com.

Many insect species with a biting-chewing feeding mechanism use their mandibles to efficiently cut through hard materials, which has provided the field of biomimetics with inspiration for synthetic devices that serve similar purposes. The studies discussed here emphasized the shape of serrations and curvature of the medial surface of mandibles (Figure 4), where the mandible interacts with food. Blade curvature is one of the most important properties of cutting efficiency [74,75], and natural selection has operated on the curvatures of mandibles to promote efficient cutting and chewing of the preferred food sources for each species. We have organized our discussion according to insect order, including Coleoptera, Orthoptera, and Hymenoptera, as these have been the main lineages employed for these studies.

##### Cutters Inspired by Coleoptera

The morphology and mechanical properties of the mandibles of beetles (Coleoptera), in particular, those of the longhorn beetles (Cerambycidae), have inspired the production of several biomimetic cutting tools. Longhorn beetles use their mandibles to bore into and consume the heartwood, branches, and stems of a variety of woody plants, causing widespread damage [76,77,78]. The mandibles of the cerambycid pine sawyer beetle, Ergates spiculatus, motivated an early biomimicry study when, in the 1940s, logger Joe Cox cut into a tree stump to reveal a tunnel created by a larva and observed its efficient cutting and chewing as it bored through the wood. He used these observations as inspiration to create a novel chainsaw chain that mimicked the mandible curvature and head movements of *E. spiculatus*. This bionic chain created cleaner cuts through the timber at a faster speed, required significantly less frequent sharpening, and is credited with revolutionizing the logging industry [79].

Other cerambycids, including adults of the common clouded longhorn beetle, Leiopus nebulosus, and the adult white-striped longhorn beetle, Batocera lineolata, had their mandible curvatures replicated to develop hemp harvest blades and alfalfa cutters, respectively (Figure 4). The biomimetic blades exhibited serration patterns that reduced the maximum cutting forces required. Both blades were found to produce smooth cuts through the stems, as compared to traditional blades, which cause visible cracking and tearing on the stem surface [78,80]. Similarly, the larvae of the bamboo weevil, Cyrtotrachelus longimanus, a member of the Curculionidae (the most diverse of all animal families with over 62,000 species) [81,82], had its mandibles studied to create blades for cutting and vegetable processing [83]. The larvae use their sharp mandibles to cut through the fibers of bamboo, a tough plant material [84], which inspired the production of two rotating bionic blades that have potential in industrial settings where conventional blades are not well-suited for vegetables with high water content and fine fibers [84].

##### Cutters Inspired by Orthoptera

The oriental migratory locust, *Locusta migratoria manilensis* (Orthoptera: Acrididae), causes widespread agricultural damage by using their specialized mandibles to cut and chew through crops and grassy plants [85,86]. Similar to some other orthopterans, *L. m. manilensis* have asymmetrical mandibles that result in a lock-and-key mechanism when chewing [87,88] (see Figure 3, for example), which likely provides efficient shearing abilities that motivated their application to bionic agricultural cutting blades. The convex-shaped incisor lobe of the mandible, as compared to a mandible with a more concave incisor, inspired the development of biomimetic straw cutters and corn stubble-cutting rotary blades for application in no-till agricultural settings [87,89]. In field trials, the bionic corn stubble-cutting blades performed better when used in pairs, which reflects the feeding mechanism of locusts [87]. Compared to traditional cutters, both sets of bionic blades reduced cutting forces and produced cleaner cuts through the stems [87,89].

Similarly, saw blades for corn stalk cutting were designed to mimic the mandibles of the citrus locust, *Chondracris rosea rosea* (Orthoptera: Acrididae), which use their mandibles to cut through fibrous cotton, reeds, and corn for subsequent ingestion [90]. Corn stalks are significant as an alternative fuel source but consist of tough fibers that are difficult to cut and harvest [91,92]. The biomimetic blades required significantly lower cutting forces and less energy to cut through the stalks. In addition, they have potential applications in harvesting other fibrous crops and in increasing agricultural energy efficiency [92].

The mandibles of crickets also have inspired several different biomimetic cutting devices. Novel-engineered cutters for tea plants and the stems of wild chrysanthemums, for example, were modeled after the contour edge of cricket mandibles. Tea plants are widely consumed and require efficient harvesting technologies, and wild chrysanthemums are valued for their medicinal properties but are difficult to efficiently harvest due to their thick stems [93,94]. The cutting edge of the mandibles of crickets was traced, extracted, and used to 3D-print resin biomimetic cutters [93,95]. These bionic blades created cleaner cuts through the stems, as compared to conventional blades that caused the stems to fray and split, and required less cutting force and energy [93,95]. Cricket mandibles also were employed for another biomimetic blade design that displayed significant improvements over traditional flat and curved blades in cutting a variety of plants, including grasses, clover, alfalfa, amaranth, nutsedge, and orach [96]. The biomimetic blade might have applications in energy conservation and sustainable agriculture.

##### Cutters Inspired by Hymenoptera

**Figure 5:**
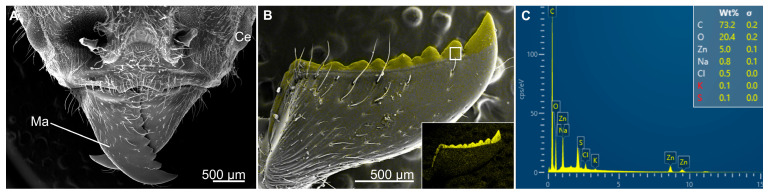
Metal presence in the cuticle of the mandibles of the leafcutter ant, Atta cephalotes (Formicidae). (**A**) SEM image of leafcutter ant head showing the mandible (Ma) and the compound eye (Ce) for reference. (**B**) Energy dispersive X-ray spectroscopy image of the mandible, showing regions of high zinc concentrations (yellow). The inset shows the same image without the SEM overlay and the white box shows where the elemental analysis was performed for image (**C**). (**C**) Output of X-ray spectroscopy showing 5% zinc in studied region.

Due to their eusocial behavior, and often accompanied tasks associated with hive or nest functionality [97,98], the mandibles of ants, bees, and wasps (Hymenoptera) also have inspired unique cutting devices. Leafcutter ants (Formicidae), for example, are well known for their metal-enriched mandibles with specialized cutting abilities [99,100] (Figure 5). The mandibles of adult leafcutter ants were used as a model to design a bionic corn root stubble-cutter and two additional biomimetic blades for harvesting the medicinal herb Chinese ginseng (*Panax notoginseng*) [101,102,103,104]. The curves of the multi-toothed mandibles were scaled up to fit industrial agricultural applications, and based on this, a serrated biomimetic blade was manufactured using laser-cutting technology. Initial testing revealed that, compared to traditional straight-edged blades, the biomimetic corn root stubble-cutter blade reduced cutting torque and power consumption [103]. The biomimetic Chinese ginseng harvest blades were found to reduce the maximum shear force required for cutting and produced more even cuts through the stems [104]. These findings suggest that the biomimetic blades are sharper, more efficient, and have potential applications in the agricultural industry to improve harvesting performance [103,104].

#### 3.2.2. Mandible-Inspired Clamps

Insect mandibles also have been applied to the design of biomimetic surgical clamps. Historically, the heads and mandibles of leafcutter ants have been used in different cultures as natural sutures [105]. External pressure would be applied to the head of the ant, forcing them to bite down on the skin, and pulling the edges of a cut or incision in the skin together. The bodies were then twisted off, leaving only the head with the mandibles clamped together, forming a natural suture [105,106]. The leafcutter ant, Atta laevigata, was used to propose a simplified biomimetic suture clamp that could be applied to and removed from the skin with ease. Modeled after their biting mechanism and mandible shape, the suggested clamps would be made of an absorbable polymer blend and require external compressive forces to open and elastic forces to clamp the skin. The surface of the clamp would mimic the microstructure of the surface of the mandibles, providing the clamp with increased elasticity and mechanical strength. Additionally, the integration of transition metals, mimicking the pattern of transition metals found within the mandibles, might provide increased strength and durability to the sutures. Future production of these mandible-inspired clamps might have important applications to human wound healing and surgical technologies [106].

#### 3.2.3. Mandible-Inspired Robotics

Goals for future technological advances in the field of robotics include the development and production of gentle-but-firm grippers at the end of mechanical arms. Grippers are essential components for many robotic manipulators; therefore, there is a need to develop grippers that are capable of carefully transporting objects, which requires a balance between using enough gripping strength to prevent dropping while avoiding material damage [107,108]. The elongated mandibles of the giant jumping ant, Harpegnathos venator (Hymenoptera: Formicidae), are used to not only clamp and secure prey, but also transport fragile eggs through narrow tunnels in the nest [109]. Therefore, their mandibles inspired the engineering of gentle gripping devices specialized for operation in narrow spaces.

Studies of mandible morphology revealed a concave region near the base that, combined with the biaxial rotation abilities, reduces contact stress and requires half as much space compared to what would be required with uniaxial rotation. Using this model, a biomimetic mechanical gripping device was designed that has potential in industrial applications that require transporting and distributing fragile objects through narrow spaces, such as supermarkets or warehouses [110]. Additional studies of the dorsal and ventral teeth of the mandibles revealed a serrated pattern that affects the friction forces and increases stability when gripping prey. Five artificial mandibles with different tooth serration patterns were 3D-printed and tested to estimate friction forces and tribological stability. It was found that the artificial mandible that most closely resembled the patterns observed on the mandibles of *H. venator* experienced the most consistent friction forces for its biaxial rotational abilities, indicating increased stability for prey handling as compared to other artificial mandibles. The biomimetic mandibles have applications as a robotic prototype for small gripping devices that could be used to manipulate and move objects of various sizes in situations where increased stability is necessary [111].

The mouthparts of insects have further motivated the development of biomimetic devices by inspiring the design of a novel robotic catapult system. The prementum of naiads of the dragonflies, *Sympetrum* sp. (Odonata: Libellulidae) and *Anax* sp. (Aeshnidae), were studied as a model for a dual catapult robotics system. Immature dragonflies and damselflies use their extendable prementum to ambush and capture prey, which is powered by a latching joint that functions as a two-link kinematic chain, creating an independently loaded, synchronized dual catapult system. Based on this, a robotics model that mimics the prementum was 3D-printed. Testing revealed that the bioinspired spring mechanism, which mimics the latching joint, was able to generate more powerful strikes when compared to the power generated by motors alone. This allows the acceleration of each segment to be independently controlled and has further applications in vector control for systems, such as jumping robots, where increased agility and maneuverability is required [112].

#### 3.2.4. Mandible-Inspired Materials

The ability to produce products with anti-adhesive surfaces is of great interest to many different disciplines and to the medical field [113,114]. Anti-adhesive surfaces prevent the attachment of bacteria, which can result in the formation of biofilms and fouling or contaminating surfaces [115]. Surfaces with anti-adhesive properties also are valuable for industrial applications to cutting surfaces, where surface contamination is problematic [116].

**Figure 6:**
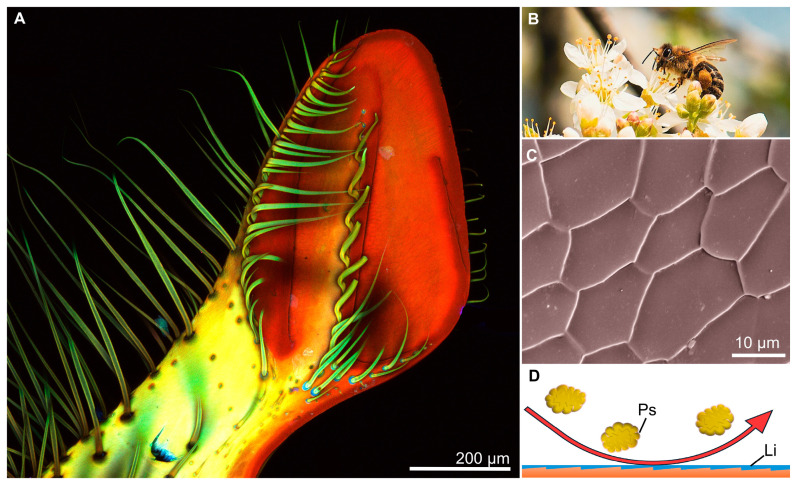
Morphology and anti-adhesive properties of the mandibles of honey bees, *Apis mellifera* (Apidae). (**A**) A confocal laser scanning microscopy (CLSM) image of the distal medial tip of the mandible of an adult honey bee, *Apis mellifera*, used to forage pollen (shown in (**B**)). (**C**) A false-colored SEM image of the microtopography and cuticle pattern on the distal medial tip of the mandibles of a honeybee. (**D**) Representative illustration of the anti-adhesive properties of the mandibles of honeybees. The uneven surface topography and thin liquid layer (Li) on the surface provide anti-adhesive properties, preventing propolis (Ps) from sticking and allowing it to slide off the mandible surface. (**B**) Image was acquired by Flocci Nivis and used under the CC Attribution-Share Alike 4.0 International license.

Insect mouthparts have inspired the development of new anti-adhesive surfaces, with the material properties and microtopography of mandibles being used as a model. The mandibles of the honey bee, *Apis mellifera* (Hymenoptera: Apidae), for example, were used in a top-down study for the development of anti-adhesive surfaces on the cutting edge of woodworking tools. Over time, resins from the wood coat the cutting edge of traditional tools, causing them to become increasingly blunt; therefore, a model organism was sought after for the development of anti-adhesive surfaces and given that the mandibles of *A. mellifera* are used to collect sticky propolis, they were further investigated [117]. Propolis is an adhesive material created out of resin, waxes, and other ingredients to build and repair hives [118]. Propolis does not adhere to the mandibles of *A. mellifera*, most likely because of a combination of a thin layer of fluid that coats the distal medial surface of the mandibles and the anisotropic, hexagonal, or pentagonal-shaped ridges that make up the surface topography [117] (Figure 6). Based on these properties, different surfaces were tested that replicate the surface topography of the mandibles of *A. mellifera*; however, it was found that without a fluid layer present, the anti-adhesive abilities were reduced [119]. Further investigations into the observed microtopography and fluid layer might have promising applications in anti-adhesive technologies.

The mandibles of insects also inspire the development of composite materials with different mechanical properties. The microstructure of the mandibles of the trap-jaw ant, Odontomachus monticola (Hymenoptera: Formicidae), for example, was used to engineer biomimetic impact-resistant composite laminates. Trap-jaw ants use their strong mandibles to strike and grab prey, reaching clamping speeds of up to 64 ms^−1^ and generating impact forces up to 300 times their body weight. The mandibles are able to withstand these forces without sustaining major cuticular damage and, therefore, were used as a model for the development of impact-resistant composite laminates. Three biomimetic carbon-fiber epoxy composites were designed using observed arrangements of the helicoidal cuticle nanofibers, each with a different degree of fiber alignment. It was found that out of the three materials tested, the composite with a 12° helicoidal-laminate structure had the most increased rigidity and was able to resist maximum contact force. This composite had a higher residual strength compared to other composites with unidirectional configurations and performed the best in dispersing impact, as the arrangement prevented cracks from spreading through the sample [120]. Additional studies added biomimetic wave patterns to the nano-fiber structures based on the patterns observed in the cuticle of *O. monticola*. Testing on six additional composite laminates with various wave ratios revealed adding waviness to the composite laminates provided a reduction in damage areas, and an increase in maximum contact force, residual strength, and energy dissipation. These biomimetic composite laminates have applications in manufacturing materials that experience high-impact forces, such as motor vehicle exteriors and aircraft wings [121].

## 4. Biomimicry of Haustellate Mouthparts of Insects

### 4.1. Overview

Insects that are classified as fluid feeders have haustellate mouthparts, where for some lineages SMIs resulted in the development of a proboscis, a feeding organ with conduits for transporting liquids from the environment to the gut. Haustellate mouthparts are found in the hemimetabolous orders Thysanoptera, Hemiptera, and Phthiraptera (Anoplura), and adults of the holometabolous orders Lepidoptera, Diptera, Hymenoptera, Trichoptera, and Siphonaptera [40,62]. As many as 50% of all insect species have haustellate mouthparts during at least one life stage [122]. Insects with haustellate mouthparts also require the action of a sucking pump in the head to generate the pressure differential for fluid transport [56,123,124] or valves to control the flow rates of incoming pressurized fluids [125]. Studies of the material properties and morphology of proboscises have revealed simultaneous fluid uptake and self-cleaning properties due to the presence of a hydrophilic-hydrophobic wetting dichotomy [47,126,127].

Insects with haustellate mouthparts can be placed into one of two groups, those that have a proboscis that requires piercing tissues for nutritive fluids (i.e., piercing-sucking feeding strategy), and those that do not have to pierce (nonpiercing-sucking feeding strategy) [62]. Insects with the nonpiercing-sucking feeding strategy feed on exposed fluids, which requires morphological adaptations to support the attainment of fluids from porous surfaces via capillary action [47,124]. Insects with the nonpiercing-sucking feeding strategy can be further categorized into the sponge-sucking and the lapping-sucking feeding strategies [62]. Insects with the sponge-sucking feeding strategy (e.g., house flies) have proboscises with spaces that facilitate the movement of fluids into internal conduits, such as the food canal, for subsequent transport to the gut. The lapping-sucking feeding mechanism similarly involves capillary action for liquid acquisition, but the liquids here are trapped in hairs on the external part of mouthparts rather than internal conduits.

### 4.2. Biomimicry of Piercing-Sucking Mouthparts

The piercing-sucking mouthparts of insects are structurally adapted for penetrating the tissues of plants, prey, or animal hosts to imbibe a liquid food source, such as xylem, phloem, hemolymph, or blood [46,128,129]. Some piercing-sucking insects, such as cicadas (Hemiptera: Cicadidae), have mouthpart cuticle augmented with transition metals to enhance the ability to penetrate hard substrates [130,131], similar to some groups of mandibulate insects [63,100,132]. There is a wide range of structural arrangements of mouthparts used to feed via puncturing, from the needle-like proboscis of the mosquito (Diptera: Culicidae) to the blades on the proboscis of horse flies (Diptera: Tabanidae), providing many opportunities for insect mouthparts to contribute to the development of fluid extraction or injection tools of multiple modalities.

Female mosquitoes, for example, use their proboscis to puncture the skin of mammal hosts to acquire a blood meal to nourish their eggs [133], which provided inspiration for the development of microneedles (small needles with a diameter of 40–100 µm) for numerous biomimetic studies [133,134,135,136,137,138]. The use of standard hypodermic needles is one of the most common invasive medical procedures and can cause discomfort or pain; however, the puncture of human skin by mosquitoes is painless due to a combination of proboscis morphology, vibration of the mouthparts during insertion, and the injection of numbing saliva [133,134,137,138]. Therefore, the mosquito proboscis and feeding mechanism can serve as inspiration in the design of painless microneedles for use in patients with aichmophobia (fear of sharp objects, including needles) [133,134,137,139].

**Figure 7:**
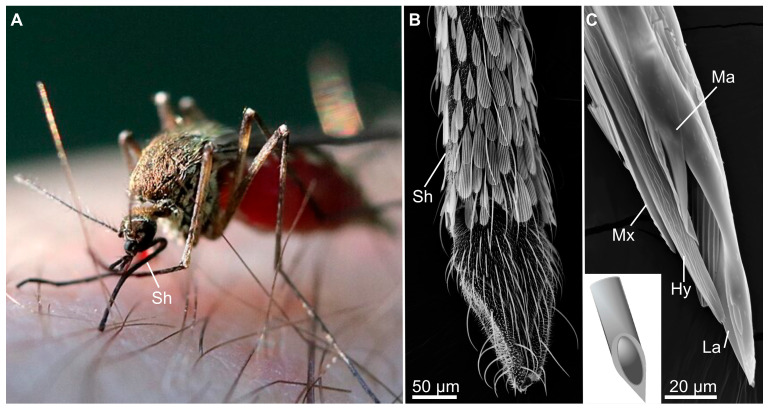
Mosquito mouthparts as a model for biomimetic needles. (**A**) A mosquito (*Ochlerotatus* sp.) using its mouthparts, which consist of six stylets and a sheath (Sh), to acquire a blood meal from a human. (**B**,**C**) SEM images of the sheath and proboscis of a mosquito (*Aedes* sp.), respectively. The proboscis is composed of the elongated maxillae (Mx), mandibles (Ma), hypopharynx (Hy), and labrum (La). The inset represents a schematic of a biomimetic needle inspired by mosquito stylets (based on [137]). (**A**) Image was acquired by Dunpharlain and used under the Creative Commons Attribution-Share Alike 4.0 International license.

Mosquitoes use a vibrating proboscis composed of a sheath, formed by the labium, that contains six stylets: the labrum, the hypopharynx, paired mandibles, and paired maxillae [133,134,135,136,137,138] (Figure 7). The stylets are similar in diameter (~50 µm) to microneedles [137]. While feeding, the labium retracts during the puncture of the skin, revealing and stabilizing the stylets [133,137,138]. The elongated mandibles and maxillae penetrate the skin via vibration (at a frequency of 6 to 30 Hz) and antiparallel movements—the sharp mandibles are used to puncture the skin, then the serrated maxillae act to anchor the proboscis in place [133,137,138,139]. The labrum is both hollow and pointed, similar to a beveled needle, and flexible so that it can bend to reach a blood vessel after insertion of the stylets [133,137]. The tip of the labrum has a lower elastic modulus and hardness than the proximal labrum; however, the vibration of the mouthparts during feeding allows the labrum to stiffen enough to successfully puncture vessels [138]. The hypopharynx acts to secrete anticoagulant and anesthetic saliva into the area to ensure continued blood flow [133,138].

Mosquito stylets act as fully functional microneedles; conversely, manufactured microneedles are subject to mechanical failure due to their small size [134,135,136,140]. Current microneedle design is simply a scaled-down traditional needle, which must be stiff enough to penetrate and resist fracture [134]. Proboscis-inspired needles have been constructed of metals, polymers, and other materials [133]. Microneedles made of silicon are stiff enough to penetrate skin, but are quite brittle, prone to fracturing, and are not approved for such use by the FDA [133,134,135]. Metal microneedles are less likely to break but can trigger allergic reactions (e.g., nickel) or must be thickened (e.g., titanium) to endure the act of puncturing the skin [133]. Polymer microneedles have less risk of fracture but do not have enough elastic modulus or hardness to puncture skin without an increase in insertion force, which could result in pain [133,134,135,136,138].

Several biomimetic studies have attempted to recreate the painless puncture achieved by the mosquito proboscis while overcoming the limitations of the material properties at the microscale. Using a softer material (e.g., polymers) to mimic the piercing labrum can make it difficult to puncture skin; however, vibrating the microneedle can decrease the insertion force required [133,138]. Adding serrated edges to mimic the shape of the maxillae further eases insertion and penetration through tissues [133,138]. A labium-like sheath can help to stretch the skin, reducing the required penetration force, and stabilize the microneedle to prevent buckling [134,136,138]. By combining several elements inspired by mosquito proboscis structure and function, a microneedle capable of penetrating the skin with little force, and, therefore, with little pain, can be manufactured. Serrated titanium and stainless-steel proboscis-inspired microneedles have been reported to successfully draw blood with little to no pain [134].

### 4.3. Biomimicry of Nonpiercing-Sucking Mouthparts

While some fluid-feeding insects must puncture tissues to access nutritive fluids, insects with nonpiercing-sucking mouthparts feed on nectar, juices on rotting fruit and carrion, and sap flows that are exposed as films, droplets, or pools on wetted surfaces [141,142,143]. The variation in the organization of mouthpart structures among the non-piercing fluid-feeding insects is extensive, but many groups have evolved proboscises that exploit capillarity to bring fluids into conduits, then a sucking pump to bring fluids to the head and gut [55,144], an excellent example of convergent evolution [47]. These mouthparts act as inspiration for biomimetic studies on devices capable of obtaining small amounts of fluids, especially from porous surfaces.

Butterflies and moths (Lepidoptera) are the most diverse group of fluid-feeding animals (over 160,000 described species) [143] and can feed on nanoscale amounts of fluids confined to small pores [47,124]. Similar to other fluid-feeding insects, Lepidoptera have a sucking pump in their heads, however, the pressure generated by the pump is not sufficient to overcome the capillary pressure holding fluids inside pores [47,55,124]. Lepidoptera overcome this obstacle by possessing a proboscis that is also porous—not a sealed, straw-like proboscis as was once believed [47,124,126]. The feeding process is further enhanced due to the presence of chemosensillae that assist in locating and identifying food sources [145,146,147].

**Figure 8:**
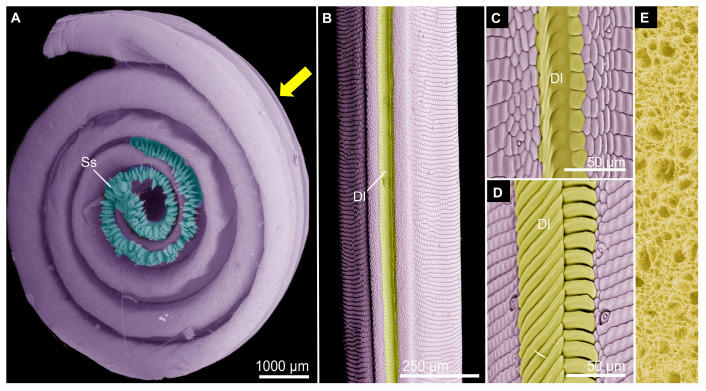
The lepidopteran proboscis as a model for microfluidic devices. (**A**) False-colored SEM image of the coiled proboscis of the red-spotted purple butterfly, *Limenitis arthemis astyanax* (Nymphalidae), which has enlarged sensilla styloconica (Ss) (false-colored blue). (**B**–**D**) SEM images of the proboscis of the monarch butterfly, *Danaus plexippus* (Nymphalidae). (**B**) The dorsal legulae (false-colored yellow, Dl) link the two c-shaped galeae (purple) together and their porosity allows liquids to travel across them via capillary action, which served as inspiration for a biomimetic fiber. (**C**,**D**) SEM images showing the porous arrangement of dorsal legulae in the nondrinking and drinking region, respectively. (**E**) Schematic of the nanopores in the fibers of a proboscis-inspired probe (based on [151]).

The lepidopteran proboscis is composed of two elongated, C-shaped maxillary galeae that join by interlinking and overlapping structures on their medial sides, the ventral and dorsal legulae, respectively, to form a circular food canal [47,148,149] (Figure 8). Pore-like interlegular spaces along the distal portion of the proboscis, i.e., the drinking region, aid the movement of fluids into the food canal via capillary action [124,126,150]. This two-level pore hierarchy (nanoscale interlegular spaces and the microscale food canal) allows the proboscis to obtain small amounts of fluids for the sucking pump to act on [151,152]. The distal drinking region of the proboscis is overall hydrophilic, due in part to the elliptical cross-section of the proboscis and hydrophilic dorsal legulae, further ensuring fluids can enter through the interlegular spaces [126,151]. The remainder of the external proboscis is hydrophobic, because of the presence of cuticular waxes and roughness of the cuticle surface; this can facilitate self-cleaning of the proboscis exterior [126,127,151].

As fluids move into the food canal of lepidopteran proboscises, a bulge forms in the liquid film on the food canal wall and eventually collapses into a liquid bridge via Rayleigh-Plateau instability, which can then be brought towards the head via the sucking pump [47,151,152]. The formation of liquid bridges inside the food canal is dependent on the relationship between the diameter of the pores from which fluids are being obtained and of the diameter of the food canal, termed the limiting pore size hypothesis—liquid bridges form when the food canal diameter is smaller than that of the liquid-filled pores [47,124].

Due to its ability to sense liquids, obtain small amounts of fluids, including those that can be viscous or sticky, and also self-clean, the lepidopteran proboscis has served as inspiration for unique microfluidic devices [150,151]. A fibrous probe, for example, was inspired by the hierarchical porosity of the lepidopteran proboscis [150]. Nanofibers were constructed by electrospinning polyvinylidene fluoride (PVDF) fibers. The PVDF polymer was bent and pulled into fibers with nanoscale pores (up to 82% porosity). PVDF is an advantageous material for this use as it is affordable and the tip can be controlled with electric fields to reach liquids. The porous nanofibers were collected, oriented, and spun into a microscale yarn. While the nanofibers themselves can act as small probes to take in fluids (Figure 8), twisting many of them into a single fiber creates a microscale hierarchy as micropores form between individual nanofibers. The nanopores within fibers take up fluid slowly, but this is overcome by the larger micropores between nanofibers in the yarn. The diameter of the resulting yarn can be fine-tuned to serve various purposes and the length is cut to resemble the form of the lepidopteran proboscis [150]. The yarn also has strong fluid-wicking abilities due to the high capillary pressure generated by the nanopores within. The resulting fibrous liquid probe can be used to handle microliters of fluids, including hazardous liquids. Additionally, the yarn can be used as a sensor due to interactions between hazardous fluids and the electric-field-induced bend of the yarn [150].

## 5. Conclusions and Future Directions

In the process of assembling this review, we found several patterns regarding biomimicry of insect mouthparts that might be of interest to researchers for future studies. In general, the two most common uses of biomimicry with insect mouthparts involved the production of cutting devices that mimic the mandible functionality of some Coleoptera, Orthoptera, and Hymenoptera [e.g., 83,87,103] and the production of piercing devices that mimic mosquito mouthparts [e.g., 133]. Although these lineages and feeding strategies are inspirational for biomimetics, there are several insect groups with unique feeding strategies that have yet to be exploited. Here, we discuss some feeding mechanisms and insect lineages that show promise for future biomimetic devices.

The biting-chewing feeding mechanism is well represented in the biomimetic literature; however, their inspiration for human-engineered devices has potential beyond cutting. Several studies of mandibles of Curculionidae have suggested applications for rotary drills and other drilling devices [153,154] and it has been suggested that the mandibles of the stag beetle, *Cyclommatus metallifer* (Coleoptera: Lucanidae), can contribute to mechanical strain sensors [155]. In addition, some mandibles have self-sharpening abilities [156,157], which has wide-ranging applications in many fields. The mandibles of the desert locust, *Schistocerca gregaria* (Orthoptera: Acrididae), for example, are augmented with transition metals [158], but only on one side; therefore, the action of moving the mandibles back and forth causes asymmetrical wear, where only one side of each mandible is worn down thus producing a sharper tip [157].

Another unrepresented feeding mechanism with possible biomimetic applications is the biting-sucking feeding mechanism. Insects with this feeding mechanism are primarily represented by Neuroptera, where the mandibulate mouthpart configuration is retained but augmented with conduits for a liquid diet [62,159]. Immature antlions (Myrmeleontidae), for example, have mandibles with a C-shaped conduit bordered by grooves and ridges that fit those of the oppositely positioned C-shaped maxilla, which together produce a circular food canal for fluids to travel through via a sucking pump in the head [59]. The antlion uses its mandibles for capturing and holding prey while the maxillae perform back-and-forth actions to pierce the cuticle to deliver venom and suck extraoral digested juices. This feeding mechanism, therefore, provides a unique combination of employing rapid, gripping abilities of the mandibles [160], alongside venom delivery and nanoscale fluid uptake mechanisms [59].

The megadiverse order Hemiptera has approximately 80,000 species, making it the largest hemimetabolous order of insects [161], but the application of their piercing-sucking feeding mechanism to biomimicry has yet to be tested. Similar to mosquitoes, hemipteran mouthparts are modified for fluid feeding [162,163], but unlike mosquitoes, most Hemiptera are not faced with the selection pressures associated with their vertebrate-blood feeding habits, such as feeding quickly and painlessly to prevent vertebrate detection [164,165,166]. Most Hemiptera feed on either plant tissues or are predators of other arthropods [167,168]; therefore, their mouthparts might not serve as the most suitable model for slender pain-free microneedles, but perhaps there are other applications, as some hemipterans, e.g., the bed bugs (Cimicidae) are specialized blood feeders [169]. Hemipteran mouthparts consist of a dual-conduit system that could inspire the development of microfluidic devices that simulate the concurrent saliva injection and fluid uptake abilities.

**Figure 9:**
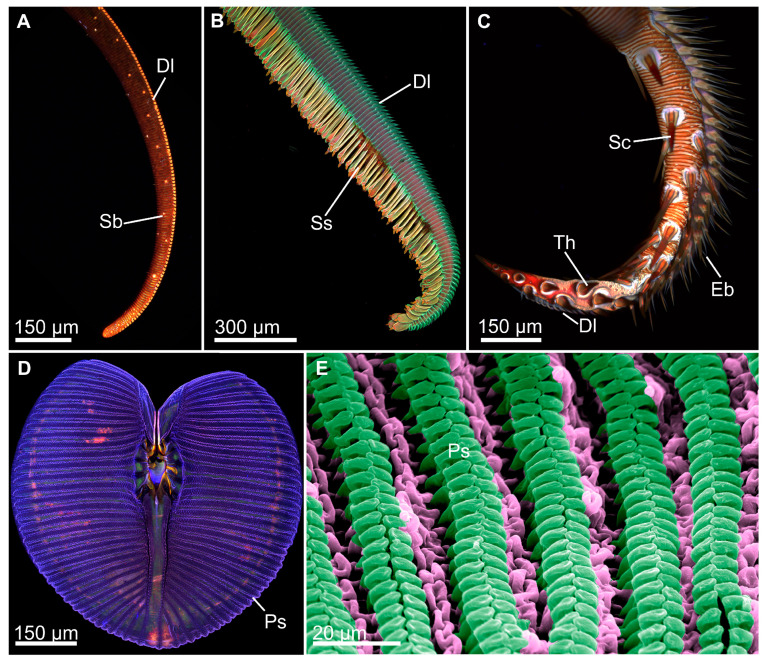
Images of the mouthparts of fluid-feeding insects with potential biomimetic applications. (**A**–**C**) Confocal laser scanning microscope (CLSM) images of the distal region of a single galea of lepidopteran proboscises. (**A**) The spicebush swallowtail, *Papilio troilus* (Papilionidae), has a smooth proboscis surface with relatively small chemosensilla (sensilla basiconica, Sb) and smooth dorsal legulae (Dl). (**B**) The proboscis of the red-spotted purple butterfly, *Limenitis arthemis astyanax* (Nymphalidae), uses its large sensilla styloconica (Ss) similar to a mop. (**C**) The proboscis of the vampire moth, *Calyptra lata* (Noctuidae), is armed with sensory cones (Sc), erectile barbs (Eb), and tearing hooks (Th) that aid in piercing tissues, such as the skin of fruit or vertebrates for a blood meal. (**D**) A CLSM image of the labellum of the house fly, *Musca domestica* (Diptera: Muscidae) that has an array of conduits, the pseudotrachea (Ps), that act similar to a sponge. (**E**) False-colored scanning electron microscope image showing the pseudotracheae (green) that are interspersed by cuticle with elastic properties (pink).

Our review revealed that the mouthparts of fluid-feeding insects that employ capillarity, particularly the megadiverse orders Lepidoptera and Diptera (sponging-sucking feeding mechanism) and Hymenoptera (lapping-sucking feeding mechanism), are not well represented in biomimetic studies, but certainly have applications for novel microfluidic devices. The lepidopteran proboscis, for example, has been applied to biomimetics, but fluid uptake by the engineered fibrous device only involves capillarity, as there is no pump. We hope that future biomimetic studies with lepidoptera proboscises will not only include a pump but also take advantage of its coiling/uncoiling abilities [170,171] and exploit the additional specializations of the distal proboscis regions that are adapted for particular feeding habits. For example, Lepidoptera that feed primarily on floral nectar are equipped with smoother, more rigid proboscises with a circular cross-section that prevents excessive bending when entering the flower, whereas those that feed on exposed viscous fluids have enlarged sensilla styloconica that give their flexible proboscis tip a mop-like morphology and functionality; vampire moths that pierce tissues for a blood meal have proboscises equipped with tearing hooks and erectile barbs to aid in the piercing actions [24,126,128] (Figure 9). The different tips could impart specific functions in the development of microfluidic devices. In addition, the lepidopteran proboscis has advanced action abilities that allow the tip to move in many directions, which also can be applied to biomimetic devices.

Many flies (Diptera) also use a sponge-sucking feeding mechanism to acquire small amounts of fluids from porous surfaces [62]. Dipteran proboscises consist of a proximal rostrum, an intermediate haustellum, and a distal labellum [40,47]. The labellum, which is applied to liquid food sources for feeding, is ornamented with an array of C-shaped conduits, the pseudotracheae, that radiate outwards from a central oral opening that leads to the food canal (Figure 9). Cuticular projections of the pseudotracheae facilitate capillary action to bring fluids into the conduits, which are channeled to the oral opening, then transported to the head by the pressure differential induced by the sucking pump [47,59]. The diameter of the pseudotracheae limits the pore size from which fluids can be obtained by the fly, suggesting liquid bridge formation within the pseudotracheae (by a similar mechanism as described in Lepidoptera), before fluids pool in the food canal and are ultimately delivered to the gut [47]. This unique, structural hierarchical organization of multiple channels with dual-functioning capillarity and suction feeding will likely inspire future advances in microfluidic devices; for example, multi-channel devices with large surface areas as opposed to the fibrous-type devices inspired by lepidopteran proboscises [150].

**Figure 10:**
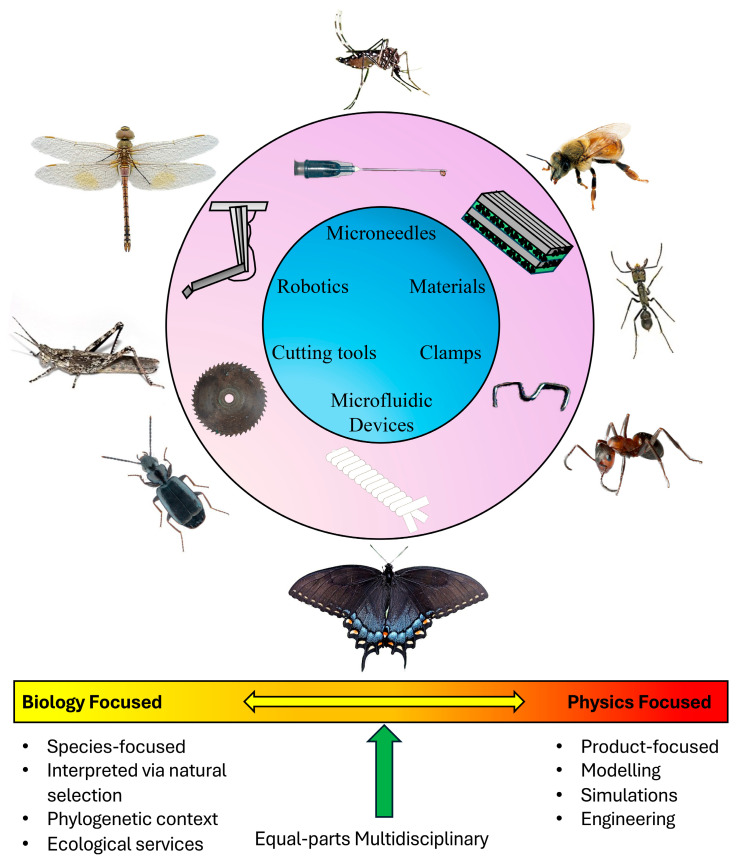
Representative diagram of patterns in biomimicry studies that feature insect mouthparts. The top image shows the main categories of tools that have been impacted by biomimetics of insect mouthparts. Outside of the circle shows representative insect orders that correspond to each tool. The bottom image is a schematic showing two ends of a spectrum in themes in biomimetics. We found that some papers were biology focused and lacked the development of a tool, whereas other papers were engineering-heavy, but lacked important biology details. Image of the mosquito (*Aedes aegypti*) (Diptera: Culicidae) was acquired by Muhammad Mahdi Karim and used under the GNU free documentation 1.2 license. The photograph of *Apis mellifera* was used under the CC attribution 3.0 unported license and acquired by CSIRO. Image of *Dinoponera quadriceps* (Hymenoptera: Formicidae) was acquired by Dider Descouens and used under the CC Attribution-Share Alike 4.0 International license. The image of an ant was used under CC attribution 2.0 generic license and acquired by Micheal Caven. The photograph of the dark morph tiger swallowtail, *Papilio glaucus* (Lepidoptera: Papilionidae) was taken by khteWisconsin and used under CC public domain. Image of the ground beetle *Microlestes minutulus* (Coleoptera: Carabidae) was used under the CC Attribution-Share Alike 4.0 International license and acquired by URSchmidt. The photograph of the bark mimicking grasshopper, *Coryphistes ruricola* (Orthoptera: Acrididae) was taken by Flagstaffotos and used under the GNU free documentation license. The image of the vagrant emperor dragonfly (*Anax ephippiger*) (Odonata: Aeshnidae) was used under the CC Attribution-Share Alike 4.0 International license and acquired by Alvesgaspar. The photograph of the needle was acquired by Nicola Sap De Mitri and used under CC Attribution Share Alike 2.0 generic license. The surgical staple clamp image was used under CC Attribution Share Alike 3.0 unported license and was acquired by Jakubtr. The yarn schematic was created by Paulo H. T. F. Alves and used under CC Attribution-Share Alike 4.0 International license. Image of the saw blade was taken by the Missouri Historical Society and used under public domain.

Insect mouthparts have inspired several different applications to the field of biomimicry (Figure 10), and we expect that improvements in technology will continue to provide new insights into the interplay between morphology and physics, and how these factors affect insect feeding mechanisms. One of the most prominent patterns we found was that publications could be placed on a continuum that represents a spectrum in the multidisciplinary nature of the studies, where one end of the continuum represents studies that primarily feature authors who are biologists, and these investigations emphasize phylogeny, morphology, and the functionality of structures from an evolutionary (adaptive) point of view, often with little physics or engineering. These papers sometimes make a general statement about how the results of the study could inspire future biomimetic research and devices. At the other end of the spectrum were manuscripts that featured primarily physicists and engineers who developed a product that was modeled after an insect mouthpart but lacked detailed information about the organism of study (we found several manuscripts that were missing the scientific name of the model organism), its phylogeny and morphology, life history traits, and why it was chosen as the model (Figure 10). We found that the most well-rounded biomimetic studies featured several authors of different specialties, including morphologists/evolutionary biologists and engineers/physicists, or consisted of authors with strong backgrounds in both subject areas. We advocate that future author assemblages for biomimicry studies represent true multidisciplinary teams that include experts from both fields, at the minimum.

## Abbreviations

The following abbreviations are used in this manuscript:

CLSM	Confocal laser scanning microscopy
SEM	Scanning electron microscopy
EDS	Energy dispersive X-ray spectroscopy
